# Are respiratory complications of *Plasmodium vivax* malaria an underestimated problem?

**DOI:** 10.1186/s12936-017-2143-y

**Published:** 2017-12-22

**Authors:** Fernando Val, Sara Avalos, André Alexandre Gomes, José Evelio Albornoz Zerpa, Gustavo Fontecha, André Machado Siqueira, Quique Bassat, Maria Graças Costa Alecrim, Wuelton Marcelo Monteiro, Marcus Vinícius Guimarães Lacerda

**Affiliations:** 1 Fundação de Medicina Tropical Dr. Heitor Vieira Dourado, Manaus, Amazonas Brazil; 2 Universidade do Estado do Amazonas, Manaus, Amazonas Brazil; 3 Microbiology Research Institute, Universidad Nacional Autónoma de Honduras, Tegucigalpa, Honduras; 4 ISGlobal, Barcelona Ctr. Int. Health Res. (CRESIB), Hospital Clínic-Universitat de Barcelona, Barcelona, Spain; 5 ICREA, Pg. Lluís Companys 23, 08010 Barcelona, Spain; 6 Instituto Nacional de Infectologia Evandro Chagas, Fundação Oswaldo Cruz, Rio de Janeiro, Brazil; 7 Pediatric Infectious Diseases Unit, Pediatrics Department, Hospital Sant Joan de Déu (University of Barcelona), Barcelona, Spain; 8 Universidad Europea de Madrid, Madrid, Spain; 9 Instituto de Pesquisas Leônidas and Maria Deane, Fundação Oswaldo Cruz, Manaus, Amazonas Brazil

**Keywords:** *Plasmodium vivax*, Severe malaria, Respiratory distress, Lung, Case fatality, Prevalence

## Abstract

**Background:**

Respiratory complications are uncommon, but often life-threatening features of *Plasmodium vivax* malaria. This study aimed to estimate the prevalence and lethality associated with such complications among *P. vivax* malaria patients in a tertiary hospital in the Western Brazilian Amazon, and to identify variables associated with severe respiratory complications, intensive care need and death. Medical records from 2009 to 2016 were reviewed aiming to identify all patients diagnosed with *P. vivax* malaria and respiratory complications. Prevalence, lethality and risk factors associated with WHO defined respiratory complications, intensive care need and death were assessed.

**Results:**

A total of 587 vivax malaria patients were hospitalized during the study period. Thirty (5.1%) developed respiratory complications. Thirteen (43.3%) developed severe respiratory complications, intensive care was required for 12 (40%) patients and 5 (16.6%) died. On admission, anaemia and thrombocytopaenia were common findings, whereas fever was unusual. Patients presented different classes of parasitaemia and six were aparasitaemic on admission. Time to respiratory complications occurred after anti-malarials administration in 18 (60%) patients and progressed very rapidly. Seventeen patients (56.7%) had comorbidities and/or concomitant conditions, which were significantly associated to higher odds of developing severe respiratory complications, need for intensive care and death (p < 0.05).

**Conclusion:**

Respiratory complications were shown to be associated with significant mortality in this population. Patients with comorbidities and/or concomitant conditions require special attention to avoid this potential life-threatening complication.

**Electronic supplementary material:**

The online version of this article (10.1186/s12936-017-2143-y) contains supplementary material, which is available to authorized users.

## Background

Malaria remains a major public health problem in endemic countries. Despite advances in control and elimination strategies, recent estimates by the World Health Organization (WHO) highlight its high morbidity and mortality. While the majority of malaria deaths are attributed to *Plasmodium falciparum*, it is now widely accepted that *Plasmodium vivax*, the most geographically widespread human malaria parasite (~ 8.5 million annual cases) may also cause severe disease, associated with 3000 annual deaths globally [1]. Despite clinical evidence of severity from the inoculation of essentially ‘non-severe’ *P. vivax* strains during malariotherapy studies during in the 1930s [2], the recognition of vivax associated severity and its associated multi-organ dysfunction is relatively new [3–8]. This historical lack of attention by the global community of malariologists may have hampered more effective policies against malaria. Factors associated with geographical dispersion of vivax infection are complex, especially regarding the prevalence of severe syndromes and fatal disease [9], with those in lower and unstable transmission settings, as in the Americas, presenting a wider variety of organ dysfunctions [10]. Underlying conditions and comorbidities have long been perceived as having a negative impact on vivax malaria outcomes. An autopsy series conducted among confirmed vivax infected individuals revealed that 76% of them presented some type of underlying condition [6]. In that study, 41% had developed lung complications [6]. However, no clear information is available regarding the general epidemiology, risk factors and prevalence of respiratory complications in vivax malaria, especially in the Brazilian Amazon.

A recent meta-analysis showed that the prevalence of acute respiratory distress syndrome (ARDS) among vivax malaria cases ranged between 2.2% (adults) and 2.8% (children), with nearly 50% mortality, confirming the significance and associated poor prognosis of this type of complication among *P. vivax* malaria patients [11]. Moreover, female sex, having any comorbidity, lower body temperature, lower haemoglobin, and low oxygen saturation values (hypoxaemia) were significantly associated with mortality. However, as only studies containing respiratory complications were included, the prevalence of respiratory complications may have been overestimated. Similarly, population-based studies are rarely reported in the literature, leading to a possible publication bias. The aims of this study were to estimate the prevalence and lethality of such complications among *P. vivax* malaria patients in the Western Brazilian Amazon and to identify variables associated with the development of severe respiratory complications according to WHO definitions, need for intensive care (ICU) and death.

## Methods

### Study site and case management

The *Fundação de Medicina Tropical Doutor Heitor Vieira Dourado* (FMT-HVD) is a tertiary care centre in Manaus (Western Brazilian Amazon) and a reference institution for infectious diseases in the north region of the country. Patients may either directly seek care at the hospital’s outpatient clinic or be referred from other local hospitals and surrounding municipalities. At hospital presentation, patients with fever, or history of fever in the preceding days, are tested for malaria by thick blood smear. Admission/arrival to the hospital may happen after microscopic diagnosis and treatment initiation at primary level of health-care. Patient data are registered in the hospital’s electronical medical charts (iDoctor^®^). Once at the hospital, all patients undergo medical consultation and a 7-day post treatment initiation follow-up visit if diagnosed with malaria. Patients presenting with any severe clinical sign according to WHO severe malaria definitions (impaired consciousness, jaundice, significant bleeding, shock, respiratory distress and others) [12] are immediately referred to the emergency department and managed accordingly. Additional investigation for concomitant infections is triggered by the presence of specific manifestations and/or at medical’s discretion through clinical investigation and routine blood and urine testing. Comorbidities are sought based on patient’s provided information and medical chart assessment. G6PD deficiency is not tested systematically.

### Malaria diagnosis and treatment

In Brazil, thick blood smears are routinely performed for malaria diagnosis and occur at different health posts in the primary level of health care. All malaria tests (positive and negative) are recorded in the national epidemiological surveillance system for malaria (SIVEP), implemented by the Brazilian National Ministry of Health in 2002. Slides are read by local microscopists and results are given following a semi-quantitative system: 1/2 + (200–300 parasites/mm^3^); 1 + (301–500 parasites/mm^3^); 2 + (501–10,000 parasites/mm^3^); 3 + (10,001–100,000 parasites/mm^3^); and 4 + (> 100,001 parasites/mm^3^). All positive slides and 10% of negative slides are routinely reviewed in a reference unit by senior microscopists. In case of divergence, the reviewed reading is updated in the SIVEP system. PCR diagnosis is not routinely performed for malaria diagnosis. Treatment is only provided after a positive smear for malaria. Anti-malarial treatment for *P. vivax* malaria occurs following WHO recommendations and Brazilian guidelines with chloroquine (CQ, 25 mg/kg divided in 3 days) for uncomplicated cases, or parenteral artemether or artesunate in patients suspected of severe malaria or severe vomiting, followed by primaquine (PQ, 3.5 mg/kg in 7 or 14 days) [13, 14].

### Study design, population and data collection

A retrospective search of charts was performed using the International Classification of Diseases aiming to identify all patients diagnosed with *P. vivax* malaria who were hospitalized. After identification, all were completely screened for respiratory complications prior to hospital presentation, upon admission or during hospitalization. These included respiratory complaints reported by the patient in the previous days to hospital presentation or at hospital presentation or by medical examination at admission or during the hospitalization period. All clinical and laboratory information was then retrieved from the patient’s medical chart using a structured questionnaire completed by two independent investigators of the study. Data regarding parasitaemia, time of diagnosis, time of treatment start and anti-malarials used were also collected if available. Fever data (axillary temperature > 37.5 °C) was also collected if available. Patients anonymity was preserved through the analysis. No restriction of age was applied.

### Respiratory complications and case definition

Respiratory distress (RD) was defined as the following: respiratory rate > 30/min and oxygen saturation < 92% on room air in adults [12] or respiratory rate > 40/min (children 12 months up to 5 years) or > 50/min (children aged from 2 to 12 months) and oxygen saturation < 90% [15]. Blood gas analysis was also used to further assist diagnosis and management when available. Both vivax and falciparum malaria share the same criteria for severe disease with exclusion of parasitaemia thresholds [12]. Severe WHO definitions for severe respiratory impairment in malaria, including acidotic breathing and pulmonary oedema (PE) were used [12]. Acute respiratory distress syndrome (ARDS) following Berlin definition was also used [16]. Definitions are presented in Table 1.

**Table 1:** Definitions used for respiratory complications associated to severe malaria

Author, year (References)	Name	Definition
WHO 2014 [12]	Severe malaria	*Respiratory distress* Rapid, deep and labored breathing (severe acidosis) Mild—sustained nasal flaring and/or mild intercostal indrawing (recession) Severe—the presence of either marked indrawing (recession) of the bony structure of the lower chest wall or deep (acidotic) breathing *Pulmonary oedema* Radiologically confirmed, or oxygen saturation < 92% on room air with a respiratory rate > 30/min, often with chest indrawing and diffuse wheeze or crepitation on pulmonary auscultation
ARDS Definition Task Force 2012 [16]	Acute respiratory distress syndrome: the Berlin definition	*Timing* Within 1 week of a known clinical insult or new or worsening respiratory symptoms *Chest imaging* (*chest radiograph or computed tomography scan*) Bilateral opacities—not fully explained by effusions, lobar/lung collapse, or nodules *Origin of oedema* Respiratory failure not fully explained by cardiac failure or fluid overload. Need objective assessment (e.g., echocardiography) to exclude hydrostatic edema if no risk factor present *Oxygenation* Mild: PaO_2_/FiO_2_ 200—300 mmHg with PEEP or CPAP ≥ 5 cm H_2_O Moderate: PaO_2_/FiO_2_ 100—200 mmHg with PEEP ≥ 5 cm H_2_O Severe: PaO_2_/FiO_2_ ≤ 100 mmHg with PEEP ≥ 5 cm H_2_O

*ARDS* acute respiratory distress syndrome,* FiO*_2_ fraction of inspired oxygen, *IQR* inter-quartile range, *PaO*_2_ arterial partial pressure of oxygen, *PEEP* positive end-expiratory pressure

### Statistical analysis

Statistical analyses were performed using independent t tests, Wilcoxon Mann–Whitney or χ^2^ tests to compare variables across groups stratified by three independent outcomes (development of severe respiratory impairment composed by patients presenting PE and/or ARDS; patients in need for intensive care support and those who died). Difference in proportions were tested by Chi squared test (corrected by Fisher’s test if necessary). Odds ratio (OR) with its 95% confidence interval (CI) was calculated for association between variables and outcomes using logistic regression. The diagnostic performance of laboratorial parameters for the three outcomes was assessed by means of receiver operating characteristic (ROC) curves. Significance was set as p < 0.05. Analyses were performed using STATA version 12.1 (Stata Corp LP, Texas USA). The Strengthening the Reporting of Observational Studies in Epidemiology (STROBE) statement was used as guidelines for reporting this study (Additional file 1) [17].

### Ethical considerations

This study was approved by the Ethics Review Board (ERB) of FMT-HVD, Manaus, Brazil (1943/2008) and the National Brazilian Committee of Ethics (CONEP) (343/2009). All data analysed were anonymous. Since data was obtained exclusively from the medical charts, the ERB gave a waiver of informed consent.

## Results

### Prevalence and lethality of respiratory complications

**Fig. 1:**
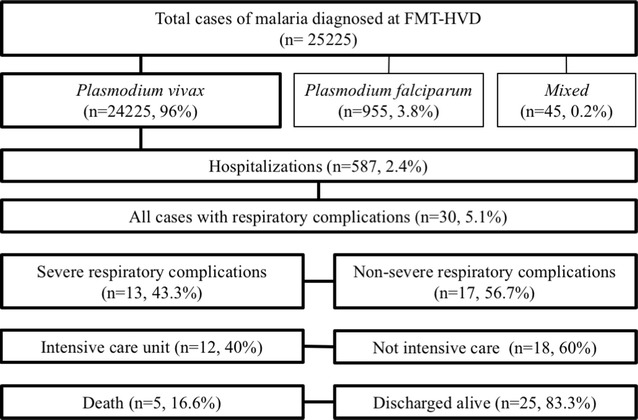
Study flow chart. Patients with *Plasmodium vivax* malaria developing respiratory complications between 2009 and 2016

From 2009 to 2016, 25,225 cases were diagnosed with malaria in the FMT-HVD, of which 24,225 (96%) were positive for *P. vivax*. A total of 587 *P. vivax* malaria patients were hospitalized. Of these, 30 (5.1%) were documented to have respiratory complications. Overall prevalence of respiratory complications was 0.1% among all vivax malaria episodes in this period. Prevalence of severe respiratory complications among hospitalized patients was 43.3% (13 patients). Twelve (40%) needed intensive care management and 5 patients (16.6%) died (Fig. 1).

### General characteristics of patients with respiratory complications

Epidemiological and clinical characteristics, treatments, parasitaemia, disease progression and outcome of all 30 cases are summarized in Table 2. Summary data is presented in Table 3. Seventeen (56.6%) patients presented previous history of malaria infections. Twenty-six (80%) patients received CQ for malaria treatment, with PQ association in 21 (70%). Fifteen (50%) patients had started anti-malarial treatment before hospitalization. Eighteen patients (60%) developed respiratory complications after anti-malarial start. Seventeen (56.6%) presented comorbidities (diabetes mellitus type 2, systemic hypertension, myasthenia gravis) and/or co-infections (HIV, *Pseudomonas aeruginosa* and *Streptococcus pneumoniae* bacteremia, detected by blood analysis). Two female patients were pregnant (Table 2). Patients with comorbidities were older than those without such conditions (45.8 ± 18.4 vs 23.7 ± 17.4, respectively; p = 0.006). G6PD deficiency was suspected after severe haemolysis associated to history of PQ treatment and was further confirmed in 5 (16.6%) patients. Haemodialysis was necessary in 5 (16.6%) patients (Table 2). Anaemia and thrombocytopaenia were common laboratory findings on admission exams (Table 2). Eleven (36.6%) patients had some degree of multi-organ involvement: 5 (16.6%) patients presented haemoglobin < 7 g/dL, four (13.3%) presented creatinine > 3 mg/dL, six (10%) presented impaired consciousness, five presented shock (16.6%) and 1 (3.3%) presented hypoglycemia (Table 2). Fifteen patients presented platelet count below 50,000/mm^3^; 12 presented platelet counts between > 50,000 and ≤ 150,000/mm^3^ and three with platelet count > 150,000/mm^3^ (Table 2).

**Table 2:** Characteristics and disease progression of patients with *P. vivax* malaria who developed respiratory complications

Id	Sex/age	Symptoms (days)	Symptoms in the previous days	Signs and symptoms at hospital presentation	Concomitant conditions/comorbidities	First malaria/number of previous episodes	Treatment previous to hospital admission/time (days)	Antimal.	Positive TBS (admission)/parasitaemia (crosses)	Respiratory complications before or after treatment initiation
1	M, 39	7	VOM (with blood), FEV, DIR	FEV, JAU, sDYS, TBC	G6PDd	No, 10	No	CQ + PQ	No, 0	After
2	F, 19	3	sHEA, FEV, CHI, VOM, ABDp, DYS	DHY, PAL, TBC	Pregnancy (22 weeks)	No, NA	No	CQ	Yes, ++	After
3	M, 58	10	FEV, CHI, dCOU, MIA, DYS	HPM, PAL, sDYS, JAU, FEV, TBC	G6PDd	NA	No	CQ	Yes, +++	Before
4	F, 50	6	FEV, CHI, HEA, ART, ABDp, DYS, dCOU	sDYS, PAL	G6PDd	No, 1	No	CQ + PQ	Yes, +	Before
5	M, 26	5	FEV, MIA, DYS, INS, CHP, ANO	FEV, TBC	HIV (bad adherence) + drug abuse	NA	No	CQ + PQ	No, 0	After
6	F, 9mo	6	FEV, CHI, CON, productive COU	DHY, PAL, DYS	Lactating	No, 1	No	CQ + PQ	Yes, ++	Before
7	M, 55	9	FEV, CHI, COU, DYS, ANO	Patient was admitted to the hospital sedated and in use of IMV. He presented PAL, DHY, TBC and generalized oedema. Patient was diagnosed with a concomitant *Streptococcus pneumoniae* (tracheal aspirate)		NA	Yes, NA	Artes.	Yes, ++	After
8	M, 23	21	FEV, CHI, MIA, VOM, HEA, ABDp, PRO, DYS	JAU, TBC	None	No, 2	Yes, 3	CQ/Artes.	Yes, ++	After
9	M, 5mo	2	FEV, DYS, HYP	PAL, sDYS, TBC	Lactating	No, 5	No	CQ	Yes, ++	Before
10	M, 46	3	CHP, COU, HEA, DYS	PAL, JAU, TBC	diabetes mellitus type 2	NA	Yes, NA	CQ + PQ	No, 0	After
11	F, 25	1	FEV, HEA, MIA, DIZ	Patient was diagnosed with vivax malaria before hospital presentation and treated with CQ + PQ + IV fluids (saline). She developed acute pulmonary oedema and was transferred to the hospital using furosemide + ATM. At admission: PAL, TBC and DYS. Patient otherwise fine, with no comorbidities		Yes	Yes, 4	CQ + PQ	No, 0	After
12	F, 47	1	EPI, FEV, JAU	Patient was diagnosed with vivax malaria before hospital presentation and treated with CQ (3 days) and PQ afterwards (incomplete—5 days). In the fifth day of PQ she developed sDYS, DIR, AST, dry COU and JAU. She than presented to hospital. Patient otherwise fine, with no comorbidities		NA	Yes, 8	CQ + PQ	Yes, ++	After
13	M, 43	4	COU, DYS, NAU	DYS, PAL, FEV, TBC	None	No, 1	No	CQ + PQ	Yes, ++	Before
14	M, 25	7	FEV, VOM, HEA	JAU, TBC	None	NA	Yes, 3	CQ + PQ	Yes, +	After
15	F, 31	10	FEV, ABDp	PAL, DHY, HYP, TBC	None	No, 1	No	CQ + PQ	Yes, +++	After
16	M, 7mo	2	FEV, DYS	PAL, TBC	Lactating	No, 1	No	CQ + PQ	Yes, ++	Before
17	F, 12	6	HEA, CHI, DIZ, NAU, VOM, DYS	DYS, DHY, FEV, TBC	None	Yes, –	No	CQ + PQ	Yes, +++	Before
18	M, 13	9	FEV, CHI, MIA, NAU, VOM, HYP, ABDp	Patient was diagnosed with vivax malaria before hospital presentation. He was treated with CQ. With clinical deterioration, patient was transferred to the hospital presenting: DYS, PAL, JAU, DIR, DHY, ABDp, TBC. Patient otherwise fine, with no comorbidities		Yes, –	Yes, NA	CQ + PQ	Yes, +	After
19	M, 46	7	FEV, CHI	Patient presented to hospital, was diagnosed with vivax malaria and received treatment (CQ + PQ) and discharged. Two days later presented again to hospital with ABDp, dCOU, sDYS, OLI, MIA, PAL, TBC. He suffered from a spleen rupture and underwent an emergency splenectomy in another hospital. Patient with diabetes mellitus type 2 and hypertension		No, 1	Yes, NA	CQ + PQ	Yes, ++	After
20	F, 72	7	INS, PRO, NAU, VOM, DIR, DSLp, sDYS	sDYS, PAL, dURI	Diabetes mellitus type 2	No, 2	Yes, NA	CQ + PQ	Yes, +	After
21	F, 65	3	ABDp, dURI, CHI, FEV, MIA, VOM, ANO	DHY, JAU, PAL, TBC	Hypertension. Patient developed ARF, DIC, SAN, SHK and coma after admission	No, 5	Yes, 3 days	CQ + PQ/artes. (ICU)	Yes, +++	After
22	F, 36	8	ABDp, CHI, COU, DYS, FEV, HEA, MIA, PAL, OLI	DHY, ACO, dURI, HPM, SPM, ANO, TBC	Pregnant	No, NA	No	Artes. (adm)	Yes, +++	Before
23	M, 43	1	CHI, DYS, FEV, HEA, MIA, PAL, OLI, VOM	ACO, PAL. Patient developed SAN, ARF, HGL, TBC and concomitant PNM (*P. aeruginosa)* and sepsis (*Staphylococcus aureus*) in the course of hospitalization	Hypertension, diabetes mellitus type 2	No, NA	Yes, 3 days	CQ + PQ	Yes, +++	After
24	M, 75	3	CHI, DYS, FEV, HEA, MIA, PAL, VOM	ACO, PAL and developed DIC, SHK, SAN, HGL, TBC. + 4 cardiopulmonary arrest episodes	Diabetes mellitus type 2	Yes	No	Artes. (adm)	Yes, ++	Before
25	M, 47	3	COU, dURI, CHI, DYS, FEV	HPM, SPM, MIA, PAL, DHY, TBC	None	Yes	Yes, 5 days	CQ + PQ	Yes, +++	After
26	M, 17	4	ABDp, CHI, DYS, FEV, HEA, DIR, MIA, PAL, VOM	PAL, SAN (PQ induced haemolysis), ARF. Patient developed HBR, SHK	G6PDd	No, NA	Yes, 5 days	CQ + PQ	No, 0	After
27	F, 41	7	ABDp, COU, dURI, CHI, DYS, FEV, MIA, VOM	PAL, JAU, ANO, TBC	None	No, NA	No	CQ	Yes, +++	After
28	F, 74	10	dURI, CHI, FEV, MIA, OLI, VOM	DHY, JAU, ARF, SHK, TBC,	diabetes mellitus type 2	No, NA	Yes, (started 3 days before ICU)	CQ + PQ	Yes, +	After
29	M, 34	9	ABDp, dURI, DYS, FEV, HEA, MIA, ANO,	ACO, PAL, DHY, JAU, ARF, HBR, SAN (PQ induced haemolysis), SHK	G6PDd	Yes	Yes, 5 days	CQ + PQ	No, 0	After
30	F, 25	4	MIA, HEA, FEV, CHI	MIA, HEA, LMS, Dysa, RD	Myasthenia Gravis	Yes, –	No	AL; Artes. (ICU)	Yes, ++	Before

Id	Sex/age	Time of appearance of respiratory complications after treatment start (days)	Type of respiratory complication/concomitant WHO severe malaria sd.	Laboratory findings at admission	Hosp. stay (days)	ICU (days)	Haemod.	Use of supplem. oxygen, time (days)/ventilator support	Outcome	
1	M, 39	After 2 days	RD/severe anaemia	Hb 6.77, Ht 18.61, RC 2.28, WC 32,520, Plat 81, got 28, gpt 31, alp 331, ggt 71, ldh 1580; bil 46, ure 194, cre 2.9	44	Yes, 42	No	Yes, 1/yes	Death	
2	F, 19	After 3 days	ARDS	Hb 7.84, Ht 21.86, RC 2.62, WC 14,590, Plat 91, got 10, gpt 5, ure 24, cre 0.6	16	Yes, 4	No	Yes, 3/yes	Discharged	
3	M, 58	–	RD/severe thrombocytopaenia	Hb 11.82, Ht 34.12, RC 4.03, WC 9980, Plat 41, got 58, gpt 99, alp 575, ggt 326, ldh 1030, bil 3.49, ure 37, cre 1	4	No, –	No	No, –	Discharged	
4	F, 50	–	RD at rest/lung crackles/intermittent dry cough	Hb 10.1, Ht 29.24, RC 3.24, WC 10,880, Plat 256, got 120, gpt 204, alp 425, ggt 416, bil 2.36, ure 28, cre 0.8	5	No, –	No	No, –	Discharged	
5	M, 26	Day 1 of hospitalization	RD/severe thrombocytopaenia	Hb 13.4, Ht 38.5, RC 4.3, WC 2500, Plat 13, got 59, gpt 25, ldh 751, ure 21, cre 1	4	No, –	No	No, –	Discharged	
6	F, 9mo	Day 1 of hospitalization	RD/cerebral manifestations (repeated convulsions)	Hb 8.08, Ht 25.15, RC 3.78, WC 10,820, Plat 146	7	No, –	No	Yes, 9/no	Discharged	
7	M, 55	NA	ARDS	Hb 8.28, Ht 24.34, RC 2.73, WC 5770, Plat 103, got 74, ggt 140, ldh 656, ure 31, cre 0.6	10	Yes, 9	Yes	Yes, 8/yes	Death	
8	M, 23	Day 1	RD/cerebral manifestations, severe thrombocytopaenia	Hb 9.83, Ht 27.98, RC 3.49, WC 740, Plat 6, got 49, gpt 27, alp 182, ggt 131, ldh 610, bil 5,61, ure 69, cre 1.2	8	Yes, 5	No	NA	Discharged	
9	M, 5mo	–	RD and nasal flaring/severe thrombocytopaenia	Hb 11.92, Ht 35.54, RC 4.61, WC 6660, Plat 4, got 37, gpt 27, alp 543, ggt 14, ldh 883, bil 0.3, ure 29, cre 0.5	6	No, –	No	Yes, 4/no	Discharged	
10	M, 46	Day 1 of hospitalization	PE	Hb 12.61, Ht 37.88, RC 4.21, WC 7140, Plat 75, got 132, gpt 104, alp 751, ggt 347, ldh 912, bil 4.7, ure 18, cre 0.7	4	No, –	No	No, –	Discharged	
11	F, 25	Same day of treatment start	CHP, DYS and PE	Hb 9.78, Ht 28.83, RC 3.41, WC 6070, Plat70, got 24, gpt 42, alp 283, ldh 1199, bil 0.47, ure 17, cre 1	3	No, –	No	No, –	Discharged	
12	F, 47	After 8 days	PE	Hb 9.45, Ht 29.19, RC 3.52, WC 11,500, Plat 506, got 50, gpt 71, alp 485, ggt 192, ldh 584, bil 0.9, ure 25, cre 0.9	4	No, –	No	No, –	Discharged	
13	M, 43	–	RD/severe thrombocytopaenia	Hb 10.43, Ht 31.03, RC 3.61, WC 5300, Plat 33, got 60, gpt 66, bil 0.4, ure 43, cre 1.3	3	No, –	No	Yes, 2/no	Discharged	
14	M, 25	After 2 days	RD+ absence of breath sounds on auscultation/severe thrombocytopaenia	Hb 11.39, Ht 34.35, RC 3,82, WC 6220, Plat 48, got 13, gpt 41, alp 206, ggt 90, ure 23, cre 1	6	No, –	No	No, –	Discharged	
15	F, 31	After 4 days	Severe RD+ central and limb cyanosis	Hb 10.09, Ht 29.15, RC 3.5, WC 6300, Plat 51, got 18, gpt 14, alp 211, ggt 124, ldh 393, bil 2.1, ure 11, cre 0.7	7	No, –	No	Yes, 7/yes	Discharged	
16	M, 7mo	Arrived with respiratory deterioration	RD+ crackles at lung auscultation/severe thrombocytopaenia	Hb 8.79, Ht 26.03, RC 3.74, WC 9540, Plat 31, got 52, gpt 15, alp 64, ldh 885, bil 0.5, ure 12, cre 0.2	11	No, –	No	No, –	Discharged	
17	F, 12	Arrived with respiratory deterioration	RD/severe thrombocytopaenia	Hb 9.78, Ht 30.29, RC 3.88, WC 3340, Plat 30, got 51, gpt 38, alp 605, ldh 901, bil 3.6, ure 25, cre 0.7	6	No, –	No	No, –	Discharged	
18	M, 13	Arrived with respiratory deterioration	RD+ crackles at lung auscultation/severe thrombocytopaenia	Hb 10.16, Ht 32.51, RC 4.14, WC 3190, Plat 28, got 38, gpt 30, alp 364, ggt 55, ldh 632, bil 3.1, ure 41, cre 0.8	5	No, –	No	No, –	Discharged	
19	M, 46	After 2 days	RD/severe anaemia, acute renal failure and severe thrombocytopaenia	Hb 5.53, Ht 15.98, RC 1.71, WC 4460, Plat 22, got 24, gpt 7, alp 134, ggt 51, ldh 563, bil 1.6, ure 96, cre 3.1	2	No, –	No	No, –	Discharged	
20	F, 72	After 4 days	PE/impaired consciousness	Hb 10.29, Ht 31.07, RC 3.39, WC 8060, Plat 101, got 50, gpt 54, alp 987, ggt 217, bil 1, ure 17, cre 1	7	Yes, 7	No	Yes, 1/no	Discharged	
21	F, 65	Arrived with respiratory complication	ARDS/acute renal failure (decreased urinary output), shock and severe thrombocytopaenia	Hb 10.4, Ht 30.9, RC 3.6, WC 1500, Plat 34, got 38, gpt 25, alp 300, ggt 359, ldh 1545, bil 12.8, ure 41, cre 1	13	Yes, 13	Yes	Yes, 13/yes	Death	
22	F, 36	Arrived with respiratory complication	RD/altered consciousness and severe thrombocytopaenia	Hb 8.1, Ht 25.15, RC 2.83, WC 4800, Plat 36, got 56, gpt 68, alp 464, ggt 53, ldh 781, bil8.5, ure 31, cre 1.2	10	No	No	Yes, 6/yes	Discharged	
23	M, 43	Arrived with respiratory complication	ARDS/acute renal failure, hypoglycemia, altered consciousness	Hb 7.6, Ht 21.7, RC 2.64, WC 12,800, Plat 66, got 45, ggt 132, ldh 627, bil 0.7, ure 116, cre 3	51	Yes, 47	Yes	Yes, 42/yes	Discharged	
24	M, 75	Arrived with respiratory complication	ARDS/severe anaemia, shock, DIC, hypoglycemia and altered consciousness	Hb 6.7, Ht 22, RC 2.5, WC 9700, Plat 79, got 94, gpt 22, ggt 36, ldh 1740, bil 0.6, ure 88, cre 2.3	3	Yes, 3	No	Yes, 3/yes	Death	
25	M, 47	Arrived with respiratory complication	RD/acute renal failure (decreased urinary output)	Hb 13, Ht 39.2, RC 4.22, WC 3900, Plat 98, got 52, ggt 51, ldh 693, bil 0.3, ure 35, cre 0.8		No	No	No	Discharged	
26	M, 17	Arrived with respiratory complication	ARDS/severe anaemia, acute renal failure and shock	Hb 5.1, Ht 13.9, RC 1.51, WC 19,600, Plat 316, got 117, gpt 29, alp 203, ggt 17, ldh 114, nil10, ure 249, cre 8	27	Yes, 14	No	Yes, 9/yes	Discharged	
27	F, 41	Arrived with respiratory complication	RD/severe thrombocytopaenia	Hb 9.9, Ht 28.3, RC 3.27, WC 3700, Plat 39, got 31, gpt 48, alp 184, ggt 79, bil 1.6, ure 31, cre 1	8	No	Yes	Yes, 3/yes	Discharged	
28	F, 74	Arrived with respiratory complication	ARDS/shock and severe thrombocytopaenia	Hb 11.7, Ht 34.1, RC 4.64, WC 6500, Plat 45, got 35, ggt 142, ldh 1389, bil 1, ure 72, cre 1.5	31	Yes, 15	No	Yes, 10/yes	Discharged	
29	M, 34	Arrived with respiratory complication	ARDS/shock and altered consciousness	Hb 6.7, Ht 18.6, RC 2.28, WC 32,500, Plat 81, got 28, ggt 71, ldh 1580, bil 26.9, ure 194, cre 3	1	Yes, 1	Yes	Yes, 1/yes	Death	
30	F, 25	Arrived with respiratory complication	PE and ARDS/severe thrombocytopaenia	Hb 11.86, Ht 34.47, RC 3.27, WC 7240, Plat 30, Cre 2.1, GOT 108	11	Yes, 11	No	Yes, 8/yes	Discharged	

*Sd.* syndromes, *FEV* fever, *sHEA* severe headache, *CHI* chills, *ABDp* abdominal pain, *DSLp* low back pain, *DYS* dyspnea, *DHY* dehydration, *PAL* pallor, *dCOU* dry cough, *MIA* muscle pain, *HPM* enlarged liver, *sDYS* severe dyspnea, *ART* arthritic pain, *INS* insomnia, *CHP* chest pain, *ANO* anorexia, *CON* convulsions, *ACO* altered consciousness, *PRO* prostration, *HYP* lack of appetite, *DIZ* dizziness, *PED* acute pulmonary oedema, *ATM* anti-malarials, *EPI* epistaxis, *AST* asthenia, *NAU* Nausea, *CYA* cyanosis, *DIR* diarrhoea, *OLI* oliguria, *SPM* spleen enlargement, *LMS* loss of muscle strength, *Dysa* dysarthria, *dURI* dark urine, *HGL* hypoglycemia, *HBR* high bilirubin levels, *RD* respiratory distress, *ARDS* acute respiratory distress syndrome, *ARF* acute renal failure, *DIC* disseminated intravascular coagulation, *SAN* severe anaemia, *PNM* pneumonia, *SHK* shock, *Hb* haemoglobin (g/dL), *Ht* haematocrit, *RC* red blood cells, *WC* white blood cells, *Plat* platelet count (× 10^3^), *NA* not available, *G6PDd* glucose 6-phosphate dehydrogenase enzyme deficiency, *TBC* thrombocytopaenia, *JAU* jaundice, *GOT* glutamic-oxaloacetic transaminase, *GPT* glutamic pyruvic transaminase, *ALP* alkaline phosphatase, *GGT* gamma-glutamyltransferase, Bil bilirubin, *Ure* urea, *Cre* creatinine, *Artes.* artesunate, *Haemod.* haemodialysis, *Hosp.* hospitalization

**Table 3:** Demographics and baseline characteristics between patients with severe and non-severe respiratory complications

Variable	All (n = 30)	Severe respiratory complications		
		No (n = 17)	Yes (n = 13)	*p* value
Age in years (mean ± SE)	36.3 (± 3.8)	28.9 (± 4.5)	45.9 (± 5.8)	0.013
Sex (m/f)	12/18	6/11	6/7	0.711
Comorbidities and concomitant conditions (n/%)	17 (56.7)	6 (35.3)	11 (84.6)	0.001
Respiratory symptoms at hospital admission (n/%)	23 (76.7)	12 (70.6)	11 (84.6)	0.427
Time of previous symptoms (days—mean ± SE)	6 (± 0.6)	6.7 (± 0.8)	5.1 (± 0.9)	0.899
Fever on admission (n/%)	12 (40)	9 (52.9)	3 (23.1)	0.141
First malaria episode (n/%)^a^	7 (23.3)	3 (17.6)	4 (30.7)	0.324
Respiratory complications after anti-malarials (n/%)	18 (60)	9 (52.9)	9 (69.2)	0.465
Anti-malarial treatment before hospitalization (n/%)	15 (50)	5 (29.4)	10 (76.9)	0.025
Patients requiring haemodialysis (n/%)	5 (16.7)	1 (5.9)	4 (30.7)	0.138
Death (n/%)	5 (16.6)	1 (5.9)	4 (30.7)	0.138
Haemoglobin (g/dL) (mean ± SE)	9.6 (± 0.4)	9.9 (± 0.5)	9.1 (± 0.6)	0.857
Leucocytes (× 10^3^/mm^3^) median (IQR)	6.5 (4.4–10.8)	5.3 (3.7–9.5)	8 (6.5–12.8)	0.042
Platelet count (× 10^3^/mm^3^) median (IQR)	49.5 (31–91)	36 (28–51)	79 (66–101)	0.016
Creatinine (mg/dL) median (IQR)^b^	1 (0.8–1.5)	0.9 (0.7–1.2)	1 (0.9–2.3)	0.280
Urea (mg/dL) median (IQR)^b^	31 (24–69)	31 (23–42)	41 (24–88)	0.442
Bilirubin (mg/dL) median (IQR)^c^	1.6 (0.7–4.7)	2.2 (0.5–3.6)	0.9 (0.7–10)	0.978
Lactate dehydrogenase (U/L) (mean ± SE)^d^	911.7 (± 91.2)	809.2 (± 86.4)	1034.6 (± 169)	0.113
AST (U/L) median (IQR)	50 (31–59)	50 (29–57)	50 (35–94)	0.583
ALT(U/L) median (IQR)	31 (25–60)	31 (35–66)	32 (25–54)	0.952
GGT (U/L) median (IQR)	107 (53–192)	75 (52–128)	141 (71–217)	0.198
Alkaline Phosphatase (U/L) (mean ± SE)^e^	384 (± 53)	329 (± 50)	501 (± 125)	0.071

Completeness of data: ^a^ 80%; ^b^ 96.6%; ^c^ 83%; ^d^ 73%; ^e^ 63.3%. Values expressed in mean (± standard error) unless stated otherwise. T-test or Wilkoxon–Mann–Whitney according to normality were used. Pearson Chi squared test was used to compare proportions. Significant if *p* < 0.05

*GGT* gama glutamil transferase, *AST* aspartate aminotransferase, *ALT* alanine aminotransferase

### Parasitaemia

Twelve patients (40%) reported having started anti-malarial treatment before hospital presentation due to positive malaria diagnosis in the previous days. A total of 6 patients (20%) were already aparasitaemic at hospital admission, 5 (16.6%) patients presented one cross parasitaemia, 11 (36.7%) presented 2 crosses and 8 (26.7%) presented 3 crosses.

### Severe respiratory complications

**Fig. 2:**
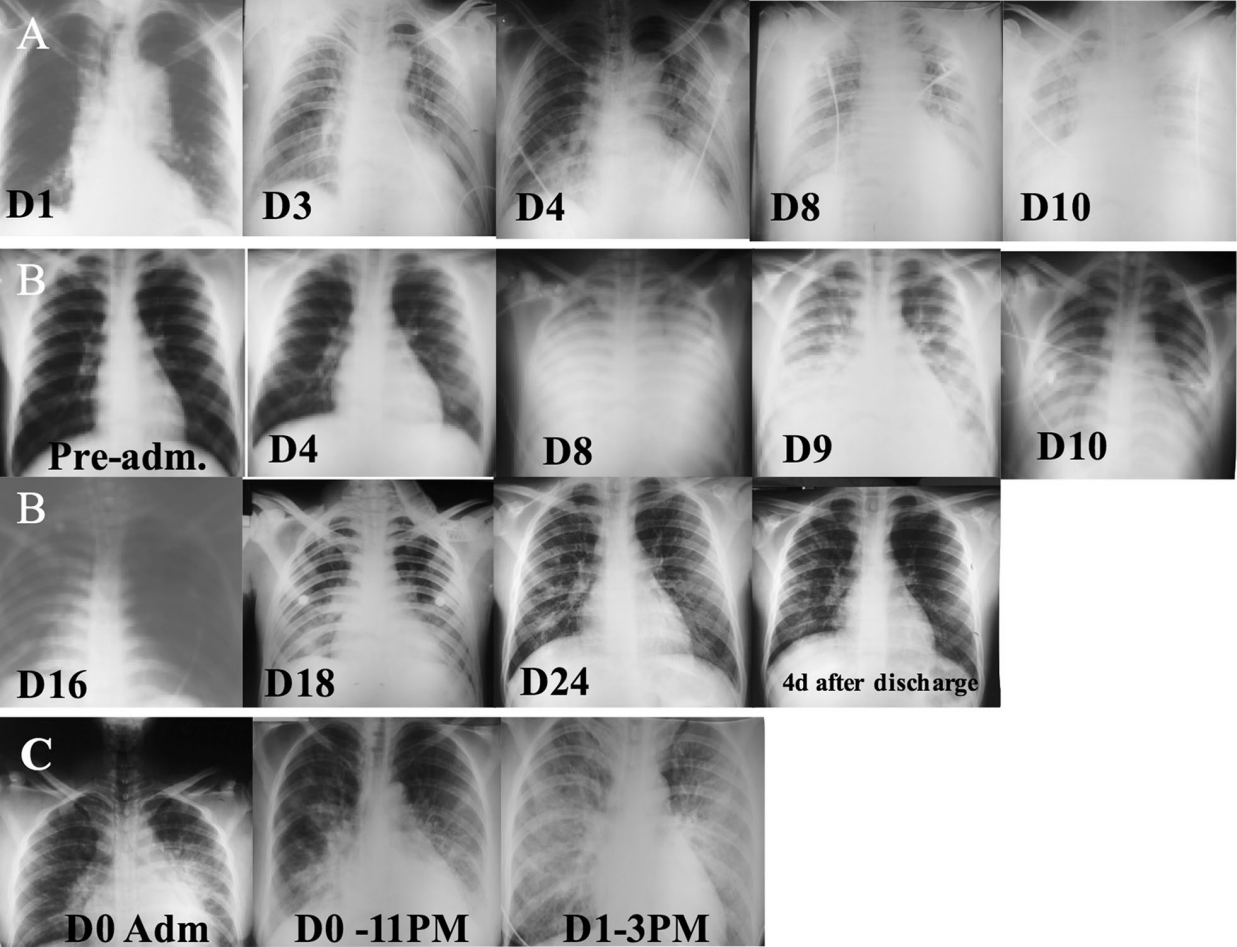
Radiological findings of three patients with *P. vivax* malaria who developed ARDS. **A** Progressed with acute renal failure, severe anaemia, disseminated intravascular coagulation and coma. Arrived intubated (D0), blood transfusion (D2), convulsions (D12), haemodialysis (D13) and died (D14). **B** Progressed with acute renal failure, hyperbilirubinemia and shock. Hospitalization (D0), intubated (D8) and discharged (D28). **C** G6PD deficient (acute haemolysis after PQ). Progressed with shock and died in the following 24 h

Of the 30 patients with a respiratory complication, 13 (43.3%) patients developed severe respiratory complications (PE and/or ARDS). The proportion of patients presenting comorbidities and/or concomitant conditions was significantly increased in this group (p = 0.001). Four patients died. Lung deterioration occurred after anti-malarials in nine patients (69.2%) of this group and progressed rapidly in some cases. Further results are presented in Table 3. Radiological findings (including alveolar infiltrates and diffuse patchy bilateral opacities) of three cases developing ARDS are presented Fig. 2.

Comorbidities and concomitant conditions [OR 9.2 (95% CI 1.3–113.2); p = 0.017] and having initiated anti-malarial treatment before hospitalization [OR 7.3 (95% 1.2–60.9); p = 0.025] were associated with higher odds of developing severe respiratory complications. Previous malaria infections, haemoglobin, platelets, leucocytes and other variables were not associated to developing severe respiratory complications in these patients. Complete results of univariate analysis for risk factors and development of severe respiratory complications are presented in Additional file 2: Table S1.

ROC analysis was performed in order to depict the discriminatory performance of laboratory variables for patients developing severe respiratory complications. Platelets [AUC 0.783 (95% CI 0.608–0.958), cut point 66000 (78.8% sensitivity; 82.3% specificity)] and leucocytes [AUC 0.781 (95% CI 0.607–0.954)] presented with reasonable discriminative performance for developing severe respiratory complications (Additional file 5: Table S4).

### Intensive care and death

Twelve (40%) patients needed intensive care support. Analysis revealed that these patients presented comorbidities and/or concomitant conditions (33.3% vs 91.6%, p = 0.002) more frequently and had lower haemoglobin values at admission (10.2 ± 0.4 g/dL vs 8.5 ± 0.6 g/dL, p = 0.003). Also, creatinine and urea at admission were found to be significantly elevated in this group, with four (33.3%, p = 0.046) patients requiring further haemodialysis (Table 4).

**Table 4:** Comparison of demographics and baseline characteristics between groups of patients in intensive care need and death outcome

Variable	Intensive care unit			Final outcome		
	No (n = 18)	Yes (n = 12)	*p*-value	Discharged alive (n = 25)	Death (n = 5)	*p*-value
Age in years (mean ± SE)	30.4 (± 4.3)	45 (± 6.4)	0.060	32.8 (± 4)	53.6 (± 7.7)	0.042
Sex (m/f)	10/8	7/5	0.880	13/12	4/1	0.249
Comorbidities and concomitant conds. (n/%)	6 (33.3)	11 (91.6)	0.002	12 (48)	5 (100)	0.032
Resp. symptoms at hospital admission (n/%)	13 (72.2)	10 (83.3)	0.481	18 (72)	5 (100)	0.177
Time of previous symptoms (days—mean ± SE)	5.4 (± 0.7)	6.2 (± 1.1)	0.501	5.6 (± 0.7)	6.2 (± 1.3)	0.740
Fever on admission (n/%)	8 (44.4)	4 (33.3)	0.543	10 (40)	2 (50)	0.315
First malaria episode (n/%)^a^	4 (30.7)	3 (27.2)	0.851	5 (25)	2 (50)	0.231
Resp. complications after anti-malarials (n/%)	13 (72.2)	10 (83.3)	0.481	18 (72)	5 (100)	0.177
Anti-malarial treatment before hospitalization (n/%)	7 (38.9)	8 (66.6)	0.264	12 (48)	3 (60)	1
Patients requiring haemodialysis (n/%)	1 (5.5)	4 (33.3)	0.046	2 (8)	3 (60)	0.004
Haemoglobin (g/dL) (mean ± SE)	10.2 (± 0.4)	8.5 (± 0.6)	0.003	9.9 (± 0.4)	7.7 (± 0.7)	0.037
Leucocytes (× 10^3^/mm^3^) median (IQR)	6.1 (3.9–9.5)	8.8 (6.1–17)	0.082	6.5 (4.4–9.9)	9.7 (5.7–32)	0.388
Platelet count (× 10^3^/mm^3^) median (IQR)	40 (30–75)	80 (39.5–96)	0.211	45 (30–91)	81 (79–81)	0.231
Creatinine (mg/dL) median (IQR)^b^	0.9 (0.7–1)	1.8 (0.9–3)	0.024	1 (0.7–1.2)	2.3 (0.9–3)	0.368
Urea (mg/dL) median (IQR)^b^	28 (21–35)	70 (36–1555)	0.004	30 (22–44)	88 (41–194)	0.034
Bilirubin (mg/dL) median (IQR)^c^	1.6 (0.5–3.5)	3.5 (1–12)	0.149	1.6 (0.7–3.5)	19.8 (6.7–36)	0.053
Lactate dehydrogenase (U/L) (mean ± SE)^d^	785.8 (± 60)	1093 (± 196)	0.098	762 (± 72)	1420 (± 194)	< 0.001
AST (U/L) median (IQR)	51 (31–58)	47 (31.5–84)	0.964	50 (33–58)	38 (28–74)	0.930
ALT(U/L) median (IQR)	41.5 (26–69)	28 (23.5–31)	0.111	38 (27–66)	25 (22–31)	0.221
GGT (U/L) median (IQR)	84 (52–259)	131 (71–142)	0.791	124 (53–192)	71 (61–140)	0.906
Alkaline Phosphatase (U/L) (mean ± SE)^e^	378 (± 54)	400 (± 149)	0.861	392 (± 60)	315 (± 15)	0.674

Completeness of data: ^a^ 80%; ^b^ 96.6%; ^c^ 83%; ^d^ 73%; ^e^ 63.3%. Values expressed in mean (± standard error) unless stated otherwise. T-test or Wilkoxon–Mann–Whitney according to normality were used. Pearson Chi squared test was used to compare proportions. Significant if *p* < 0.05

*GGT* gama glutamil transferase, *AST* aspartate aminotransferase, *ALT* alanine aminotransferase

The group composed by patients who died (n = 5, 16.6%) was older (32 ± 4 vs 54 ± 8, p = 0.042). Prevalence of comorbidities and/or concomitant conditions was significant (48% vs 100%, p = 0.032). Lower levels of haemoglobin (p = 0.037), higher levels of urea (p = 0034) and lactate dehydrogenase enzyme (p < 0.001) and also a higher proportion of patients (three, 60%, p = 0.004) requiring haemodialysis was observed. Results are presented in Table 4.

Comorbidities and/or concomitant conditions [OR 19.6 (95% CI 2–1023); p = 0.003] and creatinine [OR 3.4 (95%1.2–13.3); p = 0.009 were significantly associated with higher odds for intensive care need (complete results are presented in Additional file 3: Table S2). Haemodialysis [OR 14.5 (95% CI 1–289.7); p = 0.021] was significantly associated to higher odds of death (complete results are presented in Additional file 4: Table S3).

Receiver operating curve analysis was performed in order to depict the discriminatory performance of baseline laboratory variables for patients that needed intensive care and for those who died. Results from ROC analysis for intensive care need outcome showed that urea [AUC 0.81 (95% CI 0.63–0.959), cut point 45 (66.7% sensitivity; 94.1% specificity)] and creatinine [AUC 0.74 (95% CI 0.54–0.95), cut point 1.5 (58.3% sensitivity; 94.1% specificity)] presented reasonable discriminative performance for this outcome. When stratification was performed for final outcome (patients discharged alive or those who died), lactate dehydrogenase [AUC 0.88 (95% CI 0.64–1), cut point 1389 (55% sensitivity; 100% specificity)] and bilirubin [AUC 0.81 (95% CI 0.43–1), cut point 12.7 (75% sensitivity; 100% specificity)] presented reasonable discriminative performance. Results are presented in Additional file 5: Table S4.

## Discussion

Data from medical charts of 30 cases of hospitalized vivax malaria patients that developed respiratory complications in a tropical disease hospital of the western Brazilian amazon, between 2009 and 2016, were collected retrospectively. Anaemia and thrombocytopaenia at admission were common findings among these patients. Comorbidities and/or concomitant conditions were present in more than half of the studied population. Severe WHO defined respiratory complications and multi-organ failure developed in several cases with intensive care being necessary. Reported anti-malarial regimen consisted mainly of CQ plus PQ with a more than half of patients developing respiratory complications after treatment start.

A pooled analysis of clinical studies since the year 1900 describing complications in vivax patients showed a 0.27% prevalence of respiratory dysfunction in reports with and without severity signs [10]. Geographical stratification has shown that prevalence estimates of respiratory complications vary according to endemic regions [11], mostly due to a complex interplay of host and vector characteristics [9]. Nonetheless, part of the variation is because of definitions used to describe these respiratory complications. In the present study, prevalence of ARDS and other severe respiratory complications were found to be equivalent to studies of similar design originating from India [18–21] and other countries from South-East Asia, the Eastern Mediterranean and the Western-Pacific regions [22–26].

The presence of comorbidities and concomitant conditions and the extent they contribute to complicated disease and death in malaria has lingered for centuries and still remains a matter of debate. A recent study from India with 511 *P. vivax* patients showed no significant difference in the presence of non-infectious comorbidities between severe and non-severe patients [27]. On the other hand, a study conducted in India and Brazil with 778 patients showed that patients with comorbidities may present higher risk of complications and death [8]. Also, four out of seven patients with respiratory complications presented comorbidities in the Brazilian autopsy series [6]. Despite evidence of association between comorbidities and severe disease, this still needs elucidation in vivax malaria. It is hypothesized that comorbidities, co-infections and other concomitant conditions may become unstable due to anaemia and fever in acute vivax infections. Thus, a concurrent acute or chronic relatively controlled condition can lead to a severe and potentially life-threatening one through hypoxia exacerbation and haemodynamic decompensation [28].

G6PDd screening is not routinely performed in the Americas for vivax malaria placing this population at a high risk for haemolysis [29]. In the present study, five patients were screened for G6PD activity according to medical discretion. Due to this, G6PD status is unknown in the other patients. The precise pathogenic mechanisms involving G6PD deficiency, primaquine-induced haemolysis and the development of respiratory complications in vivax malaria deserves more research and elucidation.

Several studies have reported cases of respiratory complications in vivax malaria after treatment [11], with a tenfold increase in the risk for developing respiratory distress [8]. It is suggested that a post-treatment inflammatory phenomenon occurs within lung microvasculature in these cases leading to respiratory impairment [30, 31]. This study has shown a possible association between treatment initiation and respiratory impairment. Importantly, chloroquine is known to have immune-modulatory proprieties [32–34], which could further prevent worsening of post-treatment inflammation and associated symptoms. Nonetheless, after taking into account increasing chloroquine-resistance, a change to artemisinin combination therapies for Plasmodium blood stage killing, which does not have an anti-inflammatory profile, could further increase the number of post-treatment inflammatory complications in vivax patients, such as respiratory distress [35]. Studies addressing this specific aspect of anti-malarial treatment switch are urgently needed.

The few published histopathological analyses in vivax malaria have revealed infiltrates of monocytes, lymphocytes, and neutrophils in pulmonary microvasculature along with phagocytosed pigment and diffuse alveolar damage [36, 37] and hyaline membrane formation [38]. Severe alveolar oedema and congestion with infiltrates containing mononuclear cells and suggestive images of adhesion of *Plasmodium*-infected red cells to lung microvasculature have also been reported [39]. In *P. vivax* malaria, cytoadhesion of parasitized red blood cells also seems to occur, but to a much lesser extent and magnitude to what can be seen in *P. falciparum* [40, 41]. Additionally, peripheral parasitaemia is generally much lower in this species, due to the preferential invasion of reticulocytes [38]. Instead the relatively much greater inflammatory and endothelial activation, as revealed by elevated cytokines and other inflammatory-inducing molecules [42–44], may be responsible for alveolar-capillary barrier loss and increased permeability [38], and is probably the basis for the particular respiratory complications in this species, as seen here.

This study has limitations that need to be addressed. The authors decided to focus on respiratory complications of *P. vivax* malaria and provide a more comprehensive description of its associated clinical manifestations in detriment of comparing epidemiological, clinical and laboratory aspects between this type of complication and other WHO-defined severe malaria syndromes. Therefore, no risk factor for developing respiratory complications in comparison to other malaria related complications were assessed. The retrospective nature of this study accounts for incomplete data collection due to a lack of prospective and detailed targeted investigation, including proper registration of respiratory rates, which may lead to underestimation of the prevalence of this complication, accurate registration of oxygenation rates and mechanical ventilation parameters, which may lead to lack of proper classification of ARDS according to the Berlin definition and also proper malaria diagnosis through molecular methods and systematic co-infection screening and comorbidity investigation. The decision to admit patients was made at the attending physician’s discretion and may have resulted in selection bias, which was minimized through adoption of WHO severe malaria criteria. A great number of patients in this study had received anti-malarial treatment prior to hospital presentation with some of them presenting aparasitaemic, or probably with declining parasitaemia, at the time of admission. This could implicate in clinical improvement at this time and may have underestimated the rate of respiratory complication. In addition, the relatively low prevalence of respiratory complications, and therefore, the small sample size in this study does not support more robust predictive analyses, which may further hamper translational implications. Furthermore, pathogenic mechanisms of respiratory deterioration development could not be explored with such a study design.

## Conclusions

Respiratory complications were found to have low prevalence among all vivax episodes in this study. Despite that, this complication was found to be a frequent clinical exacerbation among *P. vivax* malaria cases in need for hospital care. Comorbidities and/or concomitant conditions occurred in older patients, were associated with worsening of cases and remained associated to elevated odds for severe respiratory complication development, intensive care need and death. In this case, age may have influenced the presence of comorbidities, and therefore, be associated to poor outcome. Nonetheless, the presence of comorbidities, but not age, directly influenced the poor outcome in this study. The results from this study points to the importance of properly recognizing the existence of complications in vivax malaria infection, especially in patients with associated comorbidities and/or concomitant conditions, and further supports rebutting the old paradigm that it does not cause important and lethal disease. Prospective studies are needed to better address this potentially dangerous clinical complication and the association between chronic, non-infectious and infectious comorbidities, severe and fatal *P. vivax* infections.

## Additional files

**Additional file 1.** STROBE Statement—checklist of items that should be included in reports of observational studies.

**Additional file 2: Table S1.** Univariate analysis for risk factors for severe respiratory complications.

**Additional file 3: Table S2.** Univariate analysis for risk factors for intensive care need.

**Additional file 4: Table S3.** Univariate analysis for risk factors for final outcome.

**Additional file 5: Table S4.** AUC of ROC depicting discriminatory performance of baseline laboratorial data according to outcome.

## Abbreviations

ARDS: acute respiratory distress syndrome
FMT-HVD: Fundação de Medicina Tropical Doutor Heitor Vieira Dourado
G6PD: glucose-6-phosphate dehydrogenase
ICU: intensive care unit
PE: pulmonary oedema
ROC: receiver operating curve
RD: respiratory distress
SIVEP: national epidemiological surveillance system for malaria
WHO: World Health Organization
