# Supplementary material for: Are respiratory complications of Plasmodium vivax malaria an underestimated problem?
Source: Malar J. 2017 Dec 22;16:495. doi: 10.1186/s12936-017-2143-y (PMC5741897; doi:10.1186/s12936-017-2143-y)
Supplement: Supplementary file 2 — Additional file 2: Table S1. Univariate analysis for risk factors for severe respiratory complications. [file 12936_2017_2143_MOESM2_ESM.docx]

Additional table S1. Univariate analysis for risk factors for severe respiratory complications.

|  | Severe respiratory complications | | | |
| --- | --- | --- | --- | --- |
| Variable | No  (n=17) | Yes  (n=13) | OR  (95% CI) | p-value |
| Age in years (mean±SE) | 28.9 (±4.5) | 45.9 (±5.8) | 1 (1-1.09) | 0.027 |
| Sex (m/f) | 6/11 | 6/7 | 0.6 (0.1-3.5) | 0.819 |
| Comorbidities and concomitant conditions (n/%) | 6((35.3) | 11(84.6) | 9.2 (1.3-113.2) | 0.017 |
| Respiratory symptoms at hospital admission (n/%) | 12 (70.6) | 11 (84.6) | 2.2 (0.2-28) | 0.651 |
| Time of previous symptoms (days - mean±SE) | 6.7 (±0.8) | 5.1 (±0.9) | 0.8 (0.6-1.1) | 0.220 |
| Fever on admission (n/%) | 9 (52.9) | 3 (23.1) | 0.3 (0.03-1.6) | 0.199 |
| First malaria episode (n/%) **^a^** | 3 (17.6) | 4 (30.7) | 2.3 (0.3-21.9) | 0.591 |
| Respiratory complications after antimalarials (n/%) | 9 (52.9) | 9 (69.2) | 1.9 (0.35-12.3) | 0.601 |
| Antimalarial treatment before hospitalization (n/%) | 5 (29.4) | 10 (76.9) | 7.3 (1.2-60.9) | 0.025 |
| Hemoglobin (g/dL) (mean±SE) | 9.9 (±0.5) | 9.1 (±0.6) | 0.8 (0.5-1.1) | 0.283 |
| Leucocytes (x10^3^/mm^3^) median (IQR) | 5.3 (3.7-9.5) | 8 (6.5-12.8) | 1 (0.99-1) | 0.214 |
| Platelet count (x10^3^/mm^3^) median (IQR) | 36 (28-51) | 79 (66-101) | 1 (0.99-1) | 0.095 |
| Creatinine (mg/dL) median (IQR)^b^ | 0.9 (0.7-1.2) | 1 (0.9-2.3) | 1.6 (0.8-4.4) | 0.144 |
| Urea (mg/dL) median (IQR)^b^ | 31 (23-42) | 41 (24-88) | 1 (0.99-1) | 0.246 |
| Bilirubin (mg/dL) median (IQR)^c^ | 2.2 (0.5-3.6) | 0.9 (0.7-10) | 0.99 (0.91-1) | 0.975 |
| Lactate dehydrogenase (U/L) (mean±SE)^d^ | 809.2 (±86.4) | 1034.6 (±169) | 1 (0.99-1) | 0.226 |
| AST (U/L) median (IQR) | 50 (29-57) | 50 (35-94) | 1 (0.99-1.04) | 0.215 |
| ALT(U/L) median (IQR) | 31 (35-66) | 32 (25-54) | 0.9 (0.97-1.01) | 0.820 |
| GGT (U/L) median (IQR) | 75 (52-128) | 141 (71-217) | 1 (0.99-1.01) | 0.406 |
| Alkaline Phosphatase (U/L) (mean±SE)^e^ | 329 (±50) | 501 (±125) | 1 (0.99-1) | 0.157 |

Abbreviations: GGT – Gama Glutamil Transferase; AST – Aspartate Aminotransferase; ALT – Alanine Aminotransferase. Completeness of data: a - 80%; b – 96.6%; c – 83%; d – 73%; e – 63.3%. Values expressed in mean (± standard error) unless stated otherwise. Exact linear regression analysis. Significant if *p* <0.05.
