# Supplementary material for: Are respiratory complications of Plasmodium vivax malaria an underestimated problem?
Source: Malar J. 2017 Dec 22;16:495. doi: 10.1186/s12936-017-2143-y (PMC5741897; doi:10.1186/s12936-017-2143-y)
Supplement: Supplementary file 3 — Additional file 3: Table S2. Univariate analysis for risk factors for intensive care need. [file 12936_2017_2143_MOESM3_ESM.docx]

Additional table S2. Univariate analysis for risk factors for intensive care need.

|  | **Intensive care unit** | | | |
| --- | --- | --- | --- | --- |
| Variable | No  (n=18) | Yes  (n=12) | OR  (95% CI) | p-value |
| Age in years (mean±SE) | 30.4(±4.3) | 45(± 6.4) | 1 (0.99-1.08) | 0.062 |
| Sex (m/f) | 10/8 | 7/5 | 1.5 (0.3-9.9) | 0.824 |
| Comorbidities and concomitant conds. (n/%) | 6(33.3) | 11(91.6) | 19.6 (2 - 1023) | 0.003 |
| Resp. symptoms at hospital admission (n/%) | 13(72.2) | 10(83.3) | 1.9 (0.24-23.7) | 0.080 |
| Time of previous symptoms (days - mean±SE) | 5.4(±0.7) | 6.2(±1.1) | 1 (0.8-1.3) | 0.845 |
| Fever on admission (n/%) | 8 (44.4) | 4 (33.3) | 0.63 (0.1-3.5) | 0.824 |
| First malaria episode (n/%) ^a^ | 4(30.7) | 3(27.2) | 0.84 (0.1-6.86) | 1 |
| Resp. complications after antimalarials (n/%) | 13(72.2) | 10(83.3) | 2.8 (0.5-22.2) | 0.323 |
| Antimal. treatment before hospitalization (n/%) | 7(38.9) | 8(66.6) | 3 (0.54-19.5) | 0.263 |
| Hemoglobin (g/dL) (mean±SE) | 10.2(±0.4) | 8.5(±0.6) | 0.67(0.43-0.9) | 0.031 |
| Leucocytes (x10^3^/mm^3^) median (IQR) | 6.1(3.9-9.5) | 8.8(6.1-17) | 1 (1 – 1.001) | 0.030 |
| Platelet count (x10^3^/mm^3^) median (IQR) | 40(30-75) | 80(39.5-96) | 1 (0.99-1) | 0.944 |
| Creatinine (mg/dL) median (IQR)^b^ | 0.9(0.7-1) | 1.8(0.9-3) | 3.4 (1.2-13.3) | 0.009 |
| Urea (mg/dL) median (IQR)^b^ | 28(21-35) | 70(36-1555) | 1.03 (1-1.08) | 0.001 |
| Bilirubin (mg/dL) median (IQR)^c^ | 1.6(0.5-3.5) | 3.5(1-12) | 1.2 (1-1.5) | 0.035 |
| Lactate dehydrogenase (U/L) (mean±SE)^d^ | 785.8(±60) | 1093(±196) | 1 (0.99-1) | 0.100 |
| AST (U/L) median (IQR) | 51(31-58) | 47(31.5-84) | 1 (0.98-1) | 0.695 |
| ALT(U/L) median (IQR) | 41.5(26-69) | 28(23.5-31) | 0.96 (0.91-1) | 0.073 |
| GGT (U/L) median (IQR) | 84(52-259) | 131(71-142) | 0.99 (0.99-1) | 0.735 |

Abbreviations: GGT – Gama Glutamil Transferase; AST – Aspartate Aminotransferase; ALT – Alanine Aminotransferase. Completeness of data: a - 80%; b – 96.6%; c – 83%; d – 73%; e – 63.3%. Values expressed in mean (± standard error) unless stated otherwise. Exact linear regression analysis. Significant if *p* <0.05.
