# Supplementary material for: Are respiratory complications of Plasmodium vivax malaria an underestimated problem?
Source: Malar J. 2017 Dec 22;16:495. doi: 10.1186/s12936-017-2143-y (PMC5741897; doi:10.1186/s12936-017-2143-y)
Supplement: Supplementary file 4 — Additional file 4: Table S3. Univariate analysis for risk factors for final outcome. [file 12936_2017_2143_MOESM4_ESM.docx]

Additional table S3. Univariate analysis for risk factors for final outcome.

|  | **Final Outcome** | | | |
| --- | --- | --- | --- | --- |
| Variable | Discharged alive (n=25) | Death (n=5) | OR  (95% CI) | p-value |
| Age in years (mean±SE) | 32.8(± 4) | 53.6(± 7.7) | 1.05 (1 – 1.12) | 0.044 |
| Sex (m/f) | 13/12 | 4/1 | 3 (0.25-168) | 0.635 |
| Comorbidities and concomitant conds. (n/%) | 12(48) | 5(100) | 6.47 (0.78-inf) | 0.052 |
| Resp. symptoms at hospital admission (n/%) | 18(72) | 5(100) | 2.35 (0.27-inf) | 0.472 |
| Time of previous symptoms (days - mean±SE) | 5.6(±0.7) | 6.2(±1.3) | 1 (0.72-1.38) | 0.948 |
| Fever on admission (n/%) | 10(40) | 2(50) | 1 (0.7-10.4) | 1 |
| First malaria episode (n/%) ^a^ | 5(25) | 2(50) | 2.8 (0.16-49.5) | 0.656 |
| Resp. complications after antimalarials (n/%) | 18(72) | 5(100) | 1 (0.09-14) | 1 |
| Antimal. treatment before hospitalization (n/%) | 12(48) | 3(60) | 1.6 (0.15-22.3) | 1 |
| Patients requiring hemodialysis (n/%) | 2(8) | 3(60) | 14.5(1-289.7) | 0.021 |
| Hemoglobin (g/dL) (mean±SE) | 9.9(±0.4) | 7.7(±0.7) | 0.6 (0.33-0.98) | 0.037 |
| Leucocytes (x10^3^/mm^3^) median (IQR) | 6.5(4.4-9.9) | 9.7(5.7-32) | 1 (1-1.001) | 0.019 |
| Platelet count (x10^3^/mm^3^) median (IQR) | 45(30-91) | 81(79-81) | 0.9 (0.9-1) | 0.942 |
| Creatinine (mg/dL) median (IQR)^b^ | 1(0.7-1.2) | 2.3(0.9-3) | 1.18 (0.7-2) | 0.409 |
| Urea (mg/dL) median (IQR)^b^ | 30(22-44) | 88(41-194) | 1 (0.99-1.02) | 0.061 |
| Bilirubin (mg/dL) median (IQR)^c^ | 1.6(0.7-3.5) | 19.8(6.7-36) | 1.2 (1 – 1.8) | 0.003 |
| Lactate dehydrogenase (U/L) (mean±SE)^d^ | 762(±72) | 1420(±194) | 1 (1 – 1.008) | 0.002 |
| AST (U/L) median (IQR) | 50(33-58) | 38(28-74) | 0.99 (0.96-1) | 0.979 |
| ALT(U/L) median (IQR) | 38(27-66) | 25(22-31) | 0.96 (0.9-1.01) | 0.305 |
| GGT (U/L) median (IQR) | 124(53-192) | 71(61-140) | 0.99 (0.98-1) | 0.930 |
| Alkaline Phosphatase (U/L) (mean±SE)^e^ | 392(±60) | 315(±15) | 0.99 (0.98-1) | 0.736 |

Abbreviations: GGT – Gama Glutamil Transferase; AST – Aspartate Aminotransferase; ALT – Alanine Aminotransferase. Completeness of data: a - 80%; b – 96.6%; c – 83%; d – 73%; e – 63.3%. Values expressed in mean (± standard error) unless stated otherwise. Exact linear regression analysis. Significant if *p* <0.05.
