# Supplementary material for: Are respiratory complications of Plasmodium vivax malaria an underestimated problem?
Source: Malar J. 2017 Dec 22;16:495. doi: 10.1186/s12936-017-2143-y (PMC5741897; doi:10.1186/s12936-017-2143-y)
Supplement: Supplementary file 5 — Additional file 5: Table S4. AUC of ROC depicting discriminatory performance of baseline laboratorial data according to outcome. [file 12936_2017_2143_MOESM5_ESM.docx]

Additional table S4. AUC of ROC depicting discriminatory performance of baseline laboratorial data according to outcome

|  | Severe respiratory complications | | Intensive care support | | Death outcome | |
| --- | --- | --- | --- | --- | --- | --- |
| Variable | AUC ROC  (95% CI) | Cut point (Sens.; Spec.) | AUC ROC  (95% CI) | Cut point (Sens.; Spec.) | AUC ROC  (95% CI) | Cut point  (Sens.; Spec) |
| Hemoglobin (g/dL) | 0.32(0.12-0.53) | 8.1 (57.1; 12.5) | 0.28(0.08-0.48) | 8.2 (50; 16.6) | 0.21(0-0.44) | 7.8 (40; 12) |
| Leuc. (x10^3^/mm^3^) | 0.78(0.61-0.95) | 7.1 (71.4; 75) | 0.68(0.46-0.91) | 12.8 (41; 100) | 0.62(0.25-0.99) | 14.5 (40; 92) |
| Plat. (x10^3^/mm^3^) | 0.78(0.61-96) | 66 (78.8; 82.3) | 0.63(0.42-0.84) | 79 (58.3; 77.8) | 0.67(0.45-0.89) | 79 (80; 72) |
| Creatinine (mg/dL) | 0.66(0.46-0.87) | 2.1 (42.8; 93.3) | 0.74(0.54-0.95) | 1.5 (58.3; 94.1) | 0.63(0.29-0.95) | 2.3 (60; 87.5) |
| Urea (mg/dL) | 0.64(0.42-0.86) | 45 (50; 86.7) | 0.81(0.63-0.99) | 45 (66.7; 94.1) | 0.80(0.61-0.99) | 88 (60; 87.5) |
| Bilirubin (mg/dL) | 0.58(0.33-0.83) | 4.7 (41.6; 84.6) | 0.67(0.43-0.91) | 4.7 (50; 86.7) | 0.81(0.43-1) | 12.7 (75; 100) |
| AST (IU/L) | 0.52(0.29-0.74) | 74 (35.7; 93.3) | 0.46(0.26-0.72) | 74 (33.3; 88.2) | 0.48(0.17-0.79) | 74 (40; 83.3) |
| ALT (IU/L) | 0.50(0.25-0.74) | 31 (60; 50) | 0.29(0.08-0.51) | 48 (12.5; 56.2) | 0.27(0.05-0.5) | 31 (33; 42.8) |
| GGT (IU/L) | 0.63(0.38-0.89) | 131 (63; 72.7) | 0.53(0.27-0.79) | 131 (60; 66.7) | 0.48(0.16-0.79) | 359 (20; 94.1) |
| DHL (U/L) | 0.70(0.45-0.95) | 912 (63.6; 91) | 0.64(0.34-0.93) | 1389 (55; 100) | 0.88(0.64-1) | 1588 (60;100) |
| AP (IU/L) | 0.65(0.38-0.92) | 283 (85.7; 50) | 0.44(0.1-0.78) | 300 (60; 42) | 0.47(0.22-0.71) | 331(50; 47) |

Sens. (Sensitivity) and Spec. (Specificity) represented in %. Abbreviations: AUC – area under the curve; ROC – receiver operating curve; CI – confidence interval; Leuc – leucocytes; Plat – platelets; AP – alkaline phosphatase.
